# Genomic Epidemiology for Estimating Pathogen Burden in a Population

**DOI:** 10.3201/eid3113.241203

**Published:** 2025-05

**Authors:** W. Tanner Porter, David M. Engelthaler, Crystal M. Hepp

**Affiliations:** Translational Genomics Research Institute, Flagstaff, Arizona, USA (W.T. Porter, D.M. Engelthaler, C.M. Hepp); Arizona State University, Phoenix, Arizona, USA (D.M. Engelthaler); Northern Arizona University, Flagstaff (C.M. Hepp)

**Keywords:** genomic epidemiology, viruses, COVID-19, SARS-CoV-2, respiratory infections, pathogen intelligence, pathogen burden

## Abstract

The role of genomics in outbreak response and pathogen surveillance has expanded and ushered in the age of pathogen intelligence. Genomic surveillance enables detection and monitoring of novel pathogens; case clusters; and markers of virulence, antimicrobial resistance, and immune escape. We can leverage pathogen genomic diversity to estimate total pathogen burden in populations and environments, which was previously challenging because of unreliable data. Pathogen genomics might allow pathogen burdens to be estimated by sequencing even a small percentage of cases. Deeper genomic epidemiology analyses require multidisciplinary collaboration to ensure accurate and actionable real-time pathogen intelligence.

The SARS-CoV-2 pandemic highlighted the importance and possibility of genomic surveillance for outbreak response and pathogen surveillance. The massive success of global SARS-CoV-2 sequencing projects, producing >17 million genomes (*1*), reflects the collective effort and dedication of the scientific and public health communities. That unparalleled dataset enabled identification of viral variants and case clusters, tracking of viral movements, and enhanced understanding of evolutionary principles. Driven by increased access to sequencing and analytic technologies, the age of pathogen intelligence has begun (*2*). That concept involves translating pathogen genomics into actionable knowledge, such as detecting outbreak clusters for transmission intervention (*3*,*4*), antimicrobial resistance markers to guide treatment (*5*), novel variants to prepare for new pandemic waves (*6*), and characterization of the evolutionary pathway of pathogens to identify mitigation opportunities (*7*). Although those applications are invaluable, modern genomics and computing power enable further expansion of genomic surveillance and the creation of large-scale pathogen intelligence.

Infectious disease trend estimation could benefit from large-scale pathogen intelligence. Case counts are often confounded by care-seeking behaviors, especially when persons experience mild illness or are asymptomatic or when diagnosis is challenging (e.g., environmental fungal diseases, such as coccidioidomycosis), leading to substantial underreporting. Statistical models can estimate undetected cases by using outside data to account for underreporting or nonreportable etiologies. However, accounting for underreporting is not a simple problem, especially when considering the role that social inequity has on reporting across space and time.

Pathogen tracking in wastewater was invaluable for proactively estimating case trends and tracking variants in near real-time across the SARS-CoV-2 pandemic. Although initially applied to sewersheds in London for tracking *Salmonella enterica* in the 1940s (*8*), the methodology continues to be extended to various pathogens. For example, wastewater surveillance for enterovirus D68, a nonreportable infection in the absence of acute flaccid paralysis, was successfully done in urban and rural communities and congregate living settings in the latter half of 2022 (D.E. Erickson et al., unpub. data, https://www.medrxiv.org/content/10.1101/2023.11.20.23297677v2). Knowledge of community-based trends for enterovirus D68 and other respiratory viruses could assist in mitigating potential albuterol shortages driven by viral-induced asthma exacerbations in children. However, wastewater surveillance is not a universal solution because accurate tracking has been less successful for organisms that are minimally shed through the gastrointestinal and urinary tracts or are highly susceptible to degradation, which results in a suboptimal genomic signal.

With increased access to sequencing data, we can expand the possibilities of pathogen intelligence and usher in a second wave of genomic epidemiology. One promising method is phylodynamics, which involves leveraging pathogen genomic diversity and estimating coalescent rates to estimate disease trends (*9*). For example, our team worked with a remote Apache community in Arizona to track a largely isolated SARS-CoV-2 outbreak in 2020 that had a public health response driven by near-complete community sampling (*4*). Linear regression showed that genomically derived effective population size estimates from 36% of cases with sequenced genomes explained 86% of the variation in total case counts over time. However, we are investigating the role that sampling bias might have had on that correlation. Nonetheless, using phylodynamic methods to estimate disease burden could be invaluable for disease surveillance, enabling targeted and cost-effective programs that use remnant or prospective samples to estimate real-time disease dynamics, on the order of days or weeks, for pathogens that measurably evolve on those timescales (*10*). The genomic, public health, and bioinformatic communities must unite to clarify how we can routinely translate pathogen genomic signals into informative transmission trends and actionable insight.

At their core, phylodynamic estimations assume that, over time, pathogens accrue mutations at a consistent rate, which enables estimation of the evolutionary trajectory and rate of coalescence. That principle defines a theoretical minimum evolutionary rate combined with genome size or sequenced region relative to a pathogen’s generation time. Previously, phylodynamic estimations were primarily confined to viral systems (*11*), where higher mutation rates, short replication periods, and large populations drive faster evolution. However, modern sequencing technologies provide larger sequenced regions, so those techniques have been used in bacterial systems (*12*) and will likely continue to expand to nonviral organisms.

In addition to evolutionary rates, the pathogen system is a critical consideration for phylodynamic inferences. In the simplest case, direct and successive human-to-human transmission enables phylodynamic estimates to be directly relatable to human disease trends (*10*,*13*); however, that model is complicated by pathogen introductions into populations and long-term infections. For pathogens with sylvatic cycles, phylodynamic estimates from nonhuman sources (e.g., vectors) reflect environmental population trends and can inform public health risks.

Sampling schemes must be considered because variations across space and time are unavoidable in most surveillance programs. Elucidating how that variation affects phylodynamic inferences and identifying optimal sampling strategies are critical for the larger community. Finally, numerous phylogenetic-based statistical models exist to conduct those analyses (*10*,*13*,*14*); however, our knowledge of how those programs perform on potentially biased or nonrepresentative datasets is limited. In addition to accuracy, computational efficiency and sustainability should be considered as genomic datasets continue to grow and require accurate and fast inferences to provide actionable insights. Large-scale multipathogen investigations are needed to compare the computational complexity, sensitivity, and specificity of phylodynamic estimates across sampling schemes, including genomic sequence subsampling and the creation of periods with increased or decreased sequencing efforts. Those analyses should benchmark findings across several phylogenetic-based statistical models and compare results to existing measures, including statistically modeled cases, because those analyses will enable the scientific and public health communities to precisely identify when phylodynamic inferences provide actionable intelligence.

In summary, genomic epidemiology will continue to transform the public health and outbreak response landscape and highlight the advantages of pathogen intelligence gathering. We have the ability and responsibility to further apply genomic principles to the public health world. That expansion of principles should involve well-characterized methods, which requires applied multidisciplinary investigations across pathogen systems and integration of real-world biases into their assessments.
